# Resolvin D1 promotes the interleukin-4-induced alternative activation in BV-2 microglial cells

**DOI:** 10.1186/1742-2094-11-72

**Published:** 2014-04-05

**Authors:** Longyan Li, Yan Wu, Yanping Wang, Jing Wu, Limin Song, Wenjing Xian, Shiying Yuan, Lei Pei, You Shang

**Affiliations:** 1 Department of Critical Care Medicine, Institute of Anesthesia and Critical Care, Union Hospital, Tongji Medical College, Huazhong University of Science and Technology, Wuhan, China; 2 Department of Neurology, Union Hospital, Tongji Medical College, Huazhong University of Science and Technology, Wuhan, China; 3 Institute of Brain Research, Huazhong University of Science and Technology, Wuhan, China; 4 Department of Anesthesiology, First Affiliated hospital of Zhengzhou University, Zhengzhou, China

**Keywords:** Resolvin D1, Microglia, Alternative activation, STAT6, PPARγ

## Abstract

**Background:**

Microglia play key roles in innate immunity, homeostasis, and neurotropic support in the central nervous system. Similar to macrophages, microglia adopt two different activation phenotypes, the classical and alternative activation. Resolvin D1 (RvD1) is considered to display potent anti-inflammatory and pro-resolving actions in inflammatory models. In this present study, we investigate the effect of RvD1 on IL-4-induced alternative activation in murine BV-2 microglial cells.

**Methods:**

BV-2 cells were incubated with RvD1 alone, IL-4 alone, or the combination of RvD1 and IL-4. Western blot and immunofluorescence were performed to detect protein levels of alternative activation markers arginase 1 (Arg1), chitinase 3-like 3 (Ym1). Moreover, we investigated the effects of RvD1 on IL-4-induced activation of signal transducer and activators of transcription 6 (STAT6) and peroxisome proliferator-activated receptor gamma (PPARγ).

**Results:**

RvD1 promoted IL-4-induced microglia alternative activation by increasing the expression of Arg1 and Ym1. RvD1 also enhanced phosphorylation of STAT6, nuclear translocation of PPARγ and the DNA binding activity of STAT6 and PPARγ. These effects were reversed by butyloxycarbonyl-Phe-Leu-Phe-Leu-Phe (a formyl peptide receptor 2 antagonist). Further, the effects of RvD1 and IL-4 on Arg1 and Ym1 were blocked by the application of leflunomide (a STAT6 inhibitor) or GW9662 (a PPARγ antagonist).

**Conclusions:**

Our studies demonstrate that RvD1 promotes IL-4-induced alternative activation via STAT6 and PPARγ signaling pathways in microglia. These findings suggest that RvD1 may have therapeutic potential for neuroinflammatory diseases.

## Background

Microglia are considered to be the resident immune cells of the central nervous system (CNS). In the normal CNS, microglia survey their surrounding microenvironment [1]. However, in a pathological state, microglia respond to environmental changes with a variety of activation states, changing both morphology and phenotype [2].

It is becoming increasingly clear that microglia, like macrophages, have at least two polarized activation states: classical and alternative activation [3-6]. Classical activation (M1) induced by lipopolysaccharide (LPS) or Th1 cytokines is associated with elevations of pro-inflammation cytokines such as IL-1, IL-6 and TNF-α, nitric oxide (NO) and reactive oxygen species (ROS), which are involved in pathogen destruction and cytotoxicity [7,8]. Alternative activation (M2) induced by Th2 cytokines IL-4, IL-13 or IL-10 is associated with elevation of arginase 1 (Arg1), chitinase 3-like 3 (Ym1), mannose receptor and found in inflammatory zone 1 (FIZZ1), which is primarily involved in tissue remodeling and healing [7,8]. Therefore, modulating microglia activation states may be a potential therapeutic targeting of neuroinflammatory diseases.

Signal transducer and activators of transcription 6 (STAT6) is activated by IL-4 and IL-13 and plays an important role in alternative activation. STAT6 is able to regulate transcription of genes typical of M2 activation [9,10]. Downstream of STAT6, peroxisome proliferator-activated receptor gamma (PPARγ) is activated in a STAT6-dependent manner [11]. PPARγ is a member of the nuclear hormone receptor superfamily and is considered to associate with lipid metabolism, oxidative metabolism, macrophage and microglia M2 polarization [12-14].

Resolvins are a family of bioactive metabolites derived from omega-3 fatty acids docosahexaenoic acid (DHA) and eicosapentaenoic acid (EPA) and display potent anti-inflammatory and pro-resolving actions in inflammatory models [15]. Resolvin D1 (7S,8R,17S-trihydroxy-4Z,9E,11E,13Z,15E,19Z-DHA, RvD1) is produced physiologically from the sequential oxygenation of DHA by 15-lipoxygenase and 5-lipoxygenase [16]. RvD1 directly activates lipoxin A4 receptor/formyl peptide receptor 2 (ALX/FPR2) and orphan receptor G protein coupling receptor 32 (GPR32), limits excessive leukocyte infiltration, attenuates the production of pro-inflammatory cytokines [17], regulates macrophage phagocytosis [18] and up-regulates pro-resolving miRNAs [19]. RvD1 can also elicite macrophage polarization toward an M2-like phenotype [20]. Previous research has indicated that another ALX/FPR2 activator, aspirin-triggered lipoxin A4, can down-regulate the LPS-induced expression of M1 markers in microglia [21-23]. However, the effect of RvD1 on microglia M2 polarization and molecular mechanisms is still unknown.

Here, we investigated the impact of RvD1 on IL-4-induced expression of M2 markers in BV-2 microglial cells and the signaling pathways involving in these processes. Our data show that RvD1 up-regulates expression of Arg1 and Ym1 in IL-4-treated microglial cells depending on STAT6 and PPARγ signaling pathways.

## Methods

### Cell culture

The immortalized murine microglia cell line BV-2 was purchased from the Cell Resource Center of Peking Union Medical College (Beijing, China) and maintained in DMEM with F12 supplement (DMEM/F12, Gibco, Grand Island, NY, USA) supplemented with 10% FBS (Gibco, Grand Island, NY, USA), 100 U/ml penicillin and 100 μg/ml streptomycin at 37°C in a humidified atmosphere of 95% air and 5% CO_2_. Before each experiment, cells were serum-starved for 12 hours. BV-2 cells were incubated with different concentrations (1 nM, 10 nM or 100 nM) of RvD1 (Cayman Chemical, Ann Arbor, MI, USA) or vehicle (0.038% ethanol) for 30 minutes before addition of 10 ng/ml murine IL-4 (PeproTech, Hamburg, Germany) under serum-free conditions. To investigate the involvement of ALX, STAT6 or PPARγ, the cells were treated with 10 μM butyloxycarbonyl-Phe-Leu-Phe-Leu-Phe (Boc-2) (GenScript Corporation, Piscataway, NJ, USA), 100 μM leflunomide (Sigma-Aldrich, St. Louis, MO, USA) or 1 μM GW9662 (Sigma-Aldrich, St. Louis, MO, USA) prior to the treatment with RvD1 for 30 minutes.

### Immunocytochemistry

BV-2 cells were cultured on sterile glass cover slips and treated according to the experimental design. Afterward, cells were fixed with 4% paraformaldehyde in PBS and permeabilized with 0.1% Triton X-100 in PBS. After rinsing, cells were blocked with 3% BSA in PBS for one hour and incubated with primary antibodies overnight at 4°C. The primary antibodies used were as follows: rabbit anti-arginase-1 (1:100, Santa Cruz, Heidelberg, Germany), Ym1 (1:50, Stem Cell Technologies, Vancouver, Canada), PPARγ (1:100, Santa Cruz, Heidelberg, Germany). After washing, cells were incubated with FITC-conjugated goat anti-rabbit IgG (1:400, Jackson Immuno Research Laboratories, West Grove, PA, USA) for one hour and counterstained with 4, 6-diamidino-2-phenylindole (DAPI, Roche, Shanghai, China) for the identification of nuclei. After washing with PBS, the cover slips were mounted with antifade mounting medium (Beyotime, Shanghai, China) on slides, and the cells were observed with an Olympus immunofluorescence microscope (Olympus, Tokyo, Japan).

### Protein extraction

For making whole cell lysates, the cells were lysed in radioimmune precipitation assay (RIPA) buffer supplemented with protease inhibitor cocktail (Roche, Shanghai, China). Nuclear and cytoplasmic fractionations were performed with NE-PER Nuclear and Cytoplasmic Extraction Reagents (Thermo Scientific, Rockford, IL, USA) according to manufacturer’s protocol.

### Western blot analysis

Equal amounts of nuclear or whole cell extracts were electrophoresed on sodium dodecyl sulfate-polyacrylamide gels, and then transferred onto a polyvinylidene difluoride membrane (Millipore, Schwalbach, Germany). The transformed membrane was blocked with 5% non-fat dry milk in Tris-buffered saline containing 0.05% Tween-20 (TBST) for one hour and incubated with primary antibodies overnight at 4°C. The primary antibodies used were as follows: rabbit anti-arginase-1 (1:500), Ym1 (1:1,000), PPARγ (1:500), phospho-Stat6 (1:500, Santa Cruz, Heidelberg, Germany), β-actin (1:1,000, Santa Cruz, Heidelberg, Germany), lamin B1 (1:1,000, Santa Cruz, Heidelberg, Germany). The membrane was washed three times with TBST for ten minutes and incubated with anti-rabbit IgG-horseradish peroxidase (1:5,000, Jackson ImmunoResearch Laboratories, West Grove, PA, USA) at room temperature for one hour. The Supersignal West Pico chemiluminescent substrate system (Millipore, Schwalbach, Germany) was used to detect immunoreactive bands. The intensity of protein bands after Western blot were quantitated by using Quantity One Version 4.6.3 Image software (Bio-Rad, Hercules, CA, USA) and normalized against proper loading controls.

### Electrophoretic mobility shift assay (EMSA)

Nuclear extracts were prepared as described above. Oligonucleotides corresponding to the STAT6 (5′-TGCCTTAGTCAACTTCCCAAGAACAGA-3′) and PPARγ (5′- GGAACTAGGTCAAAGGTCATCCCCT-3′) binding site consensus sequences were synthesized and end-labeled with biotin by Invitrogen (Invitrogen, Shanghai, China). EMSAs were performed using the LightShift chemiluminescent EMSA kit (Thermo Scientific, Rockford, IL, USA). Briefly, 10 fmol of biotin-labeled, double strand probe were incubated for 20 minutes at room temperature in 20 μl of EMSA binding buffer containing 2.5% glycerol, 5 mM MgCl_2_, 50 ng/μl poly (dI-dC), 0.05% Nonidet P-40, and 6 μg of nuclear proteins. For competition EMSA, 200-fold (2 pmol) excess unlabeled, double strand probe was added to the binding reaction. The DNA-nuclear protein complexes were resolved by electrophoresis in 6% nondenaturing polyacrylamide gel in 0.5 × Tris-borate-EDTA (TBE) buffer at 100 V. Gels were then electroblotted onto Hybond nylon membranes (GE Healthcare, Freiburg, Germany) at 380 mA for 50 minutes. The membranes were then cross-linked for 15 minutes with the membrane face down on a transilluminator at 312 nm, and the biotinylated protein-DNA bands were detected with HRP-conjugated streptavidin using the chemiluminescent nucleic acid detection system.

### Statistical analysis

Data are expressed as means ± SEM of the indicated number of independent experiments. Statistical significance between multiple groups was analyzed by one-way ANOVA. Least significant difference (LSD) *post hoc* test was used for multiple comparisons. Statistical analysis was performed using the SPSS software version 13.0 (SPSS Inc., Chicago, IL, USA). *P* < 0.05 was considered statistically significant.

## Results

### RvD1 promotes IL-4-induced alternative activation of BV-2 microglial cells

**Figure 1:**
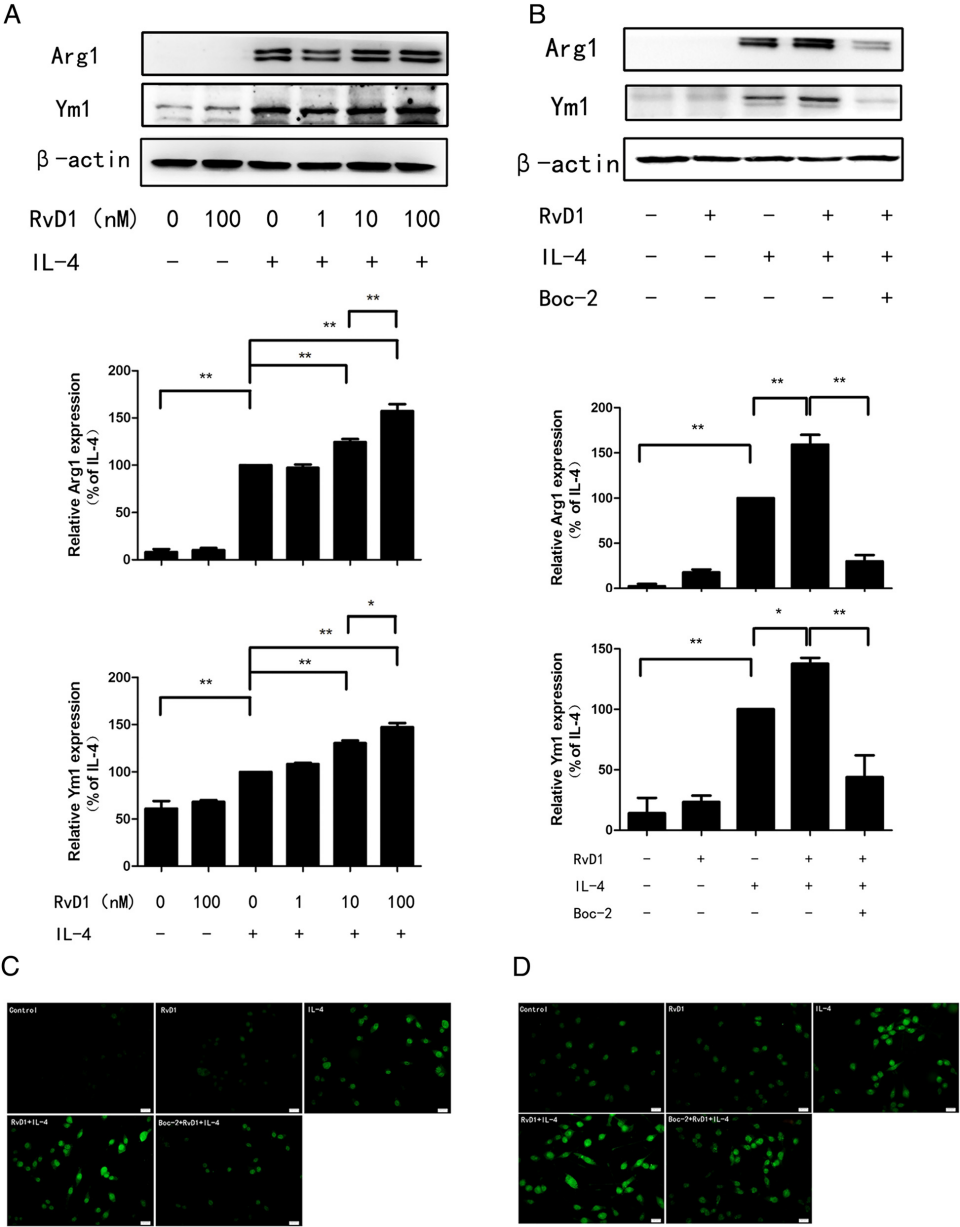
**RvD1 promotes the IL-4-induced alternative activation of BV-2 cells.** BV-2 cells were treated with vehicle (0.038% ethanol) or different concentrations of RvD1 (1 nM, 10 nM and 100 nM) for 30 minutes in the absence or presence of Boc-2 (10 μM), and then stimulated by IL-4 (10 ng/ml) for 24 hours. **(A, B)** The expression of Arg1 and Ym1 proteins was assessed by Western blot. A representative result from three independent experiments is shown. Quantification for Arg1 and Ym1 was normalized by β-actin. Data are presented as mean ± SEM for three independent experiments. Asterisks indicate statistically significant difference (*P < 0.05, **P < 0.01). **(C, D)** Representative images of BV-2 cells with positive immunofluorescence staining for Arg1 **(C)** and Ym1 **(D)**. Scale bars indicate 20 μm.

Initially, we evaluated the effects of RvD1 on IL-4-induced microglia alternative activation; BV-2 cells were treated with vehicle or different concentrations of RvD1 (1 nM, 10 nM and 100 nM) for 30 minutes and stimulated with IL-4 (10 ng/ml) for 24 hours. As the assessment of the alternative activation relied on the molecule markers such as Arg1 and Ym1, the expression of Arg1 and Ym1 were analyzed. We tested the protein levels of Arg1 and Ym1 using Western blot analysis. As shown in Figure 1A, IL-4 stimulation significantly enhanced Arg1 and Ym1 protein levels in BV-2 cells (*P* <0.01). Treatment with RvD1 alone did not affect Arg1 and Ym1 protein levels (*P* > 0.05); while combination of RvD1 and IL-4 markedly increased expression of Arg1 and Ym1 in BV-2 cells compared to IL-4 treatment alone, the effects of RvD1 were concentration dependent. Immunofluorescence staining demonstrated that Arg1 and Ym1 staining intensity increased after IL-4 treatment, pretreatment with RvD1 further increased the staining intensity (Figure 1C and D).

To evaluate the role of ALX/FPR2 in the effects of RvD1, an ALX antagonist Boc-2 (10 μM) was administrated 30 minutes prior to treatment with RvD1. The effects in response to RvD1 were reversed by Boc-2 (Figure 1B-D).

### RvD1 promotes IL-4-induced STAT6 phosphorylation and activation

**Figure 2:**
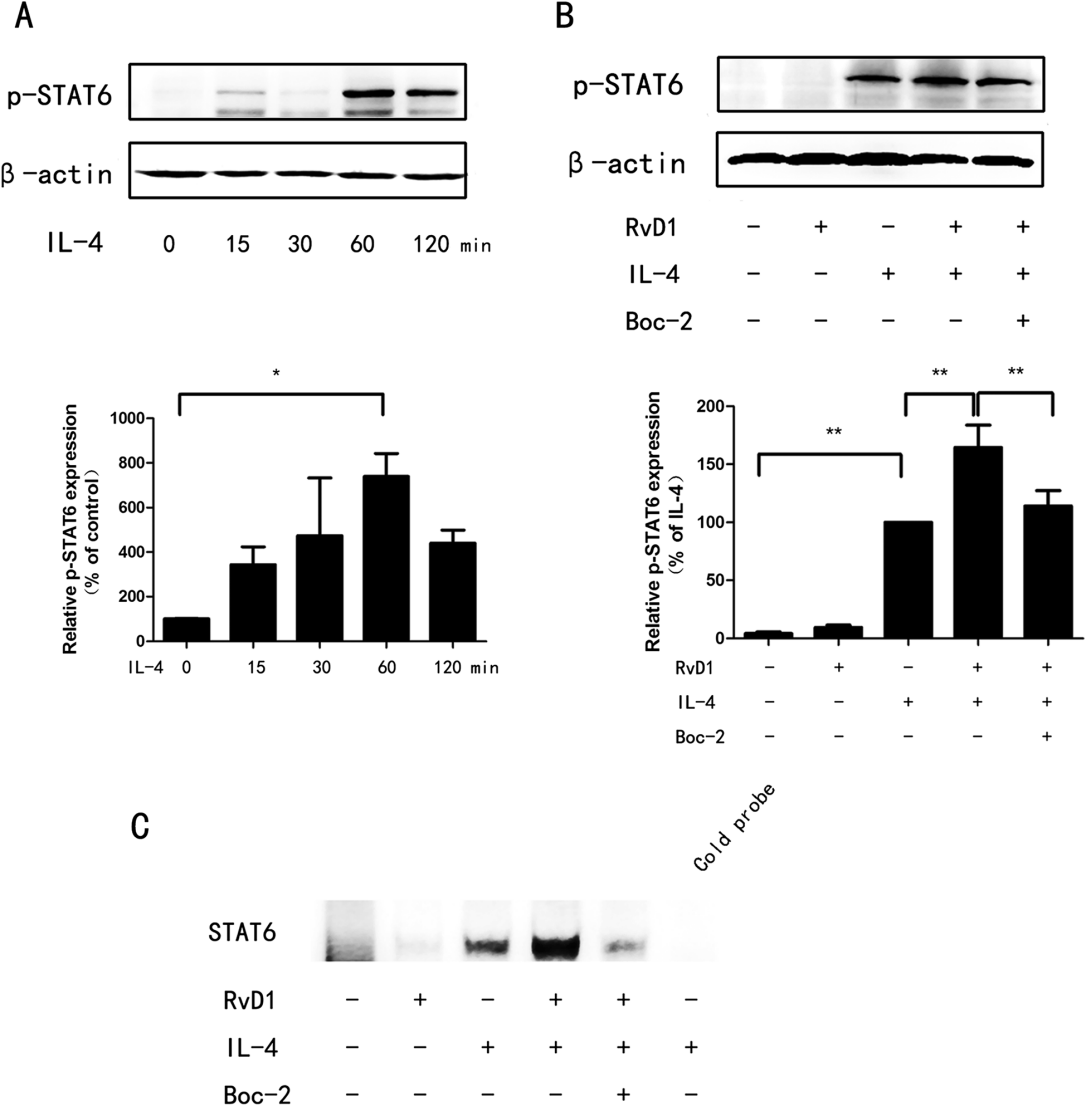
**RvD1 enhances IL-4-induced STAT6 activation. (A)** BV-2 cells were treated with IL-4 (10 ng/ml) for the indicated times, expression of phosphorylated STAT6 was assessed by Western blot. Quantification of phospo-STAT6 from three independent experiments indicated that the effect of IL-4 was obvious at 60 minutes. **(B)** BV-2 cells were treated with RvD1 (100 nM), IL-4 (10 ng/ml), Boc-2 (10 μM) or combination of them. Levels of phosphorylated STAT6 were detected by Western blot 60 minutes after IL-4 stimulation. A representative result from three independent experiments is shown. Quantification of phosphorylated STAT6 was normalized by β-actin. **(C)** BV-2 cells were treated as in **(B)**. Nuclear extracts were prepared 120 minutes after IL-4 treatment and used to analyze STAT6 DNA binding activity by Electrophoretic Mobility Shift Assay (EMSA). Binding specificity was confirmed by unlabelled probe (200-fold in excess) to compete with labeled oligonucleotide. Results were confirmed by three independent experiments. Data are presented as mean ± SEM for three independent experiments. Asterisks indicate statistically significant difference (*P < 0.05, **P < 0.01).

STAT6 plays a critical role in IL-4-dependent induction of Arg1 and Ym1 [24,25]. As RvD1 promotes IL-4-induced expression of Arg1 and Ym1 (Figure 1), we investigated the involvement of STAT6 activation in these effects. STAT6 phosphorylated at 60 minutes after IL-4 treated (Figure 2A), the 60 minute treatment of IL-4 was determined to examine the effect of RvD1 on STAT6 phosphorylation. RvD1 markedly enhanced IL-4-induced phosphorylation of STAT6 (*P* < 0.01), the effect of RvD1 was reversed by Boc-2 (*P* <0.05, Figure 2B). Further, we performed EMSA to determine the effects RvD1 and IL-4 on STAT6 DNA binding activity in BV-2 cells. IL-4 treatment increased STAT6 binding activity, combination of RvD1 and IL-4 further increased the activity. Moreover, treatment with Boc-2 reversed the effect of RvD1 (Figure 2C).

### RvD1 promotes IL-4-induced PPARγ nuclear translocation and activation

**Figure 3:**
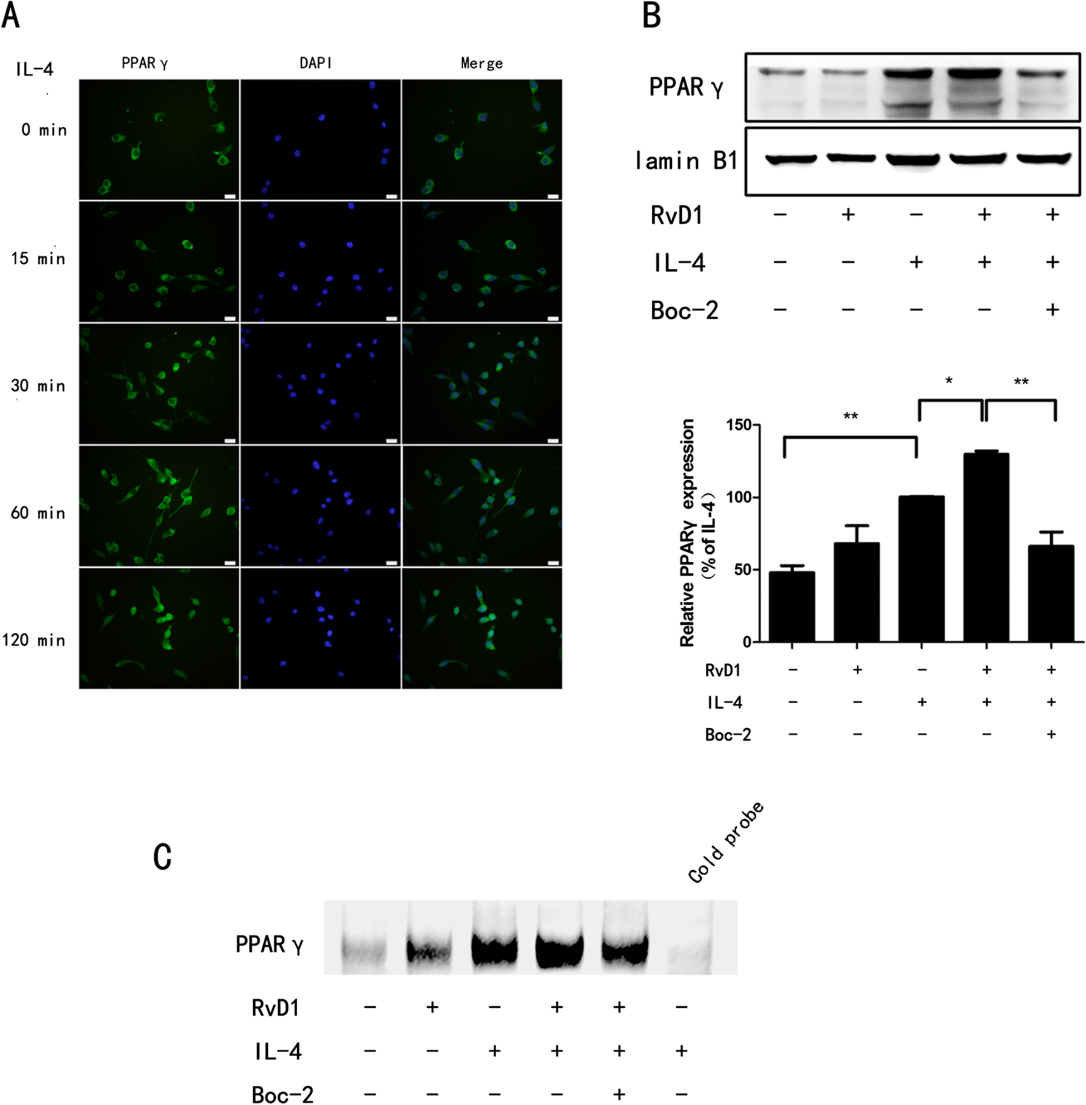
**RvD1 enhances IL-4-induced PPARγ activation. (A)** BV-2 cells were treated with IL-4 (10 ng/ml) for the indicated times, subcellular localization of PPARγ was evaluated using immunofluorescence staining. DNA was stained using 6-diamidino-2-phenylindole (DAPI) to visualize nuclei. The nuclear translocation of PPARγ was obvious 120 minutes after stimulation. Scale bars indicate 20 μm. **(B, C)** BV-2 cells were treated with RvD1 (100 nM), IL-4 (10 ng/ml), Boc-2 (10 μM) or a combination of them. Nuclear extracts were prepared 120 minutes after IL-4 treatment, Western blot or electrophoretic mobility shift assay (EMSA) were performed to detect PPARγ protein level in nucleus **(B)** or DNA binding activity **(C)**. A representative result from three independent experiments is shown. Quantification of PPARγ was normalized by lamin B1. Data are presented as mean ± SEM for three independent experiments. Asterisks indicate statistically significant difference (*P < 0.05, **P < 0.01).

Along with STAT6, PPARγ is known to control genes associated with alternatively activation [12,26]. Therefore, we examined the effects of RvD1 on IL-4-stimulated PPARγ nuclear translocation and DNA binding activity in BV-2 cells. Immunofluorescence staining indicated that PPARγ translocated from the cytoplasm into the nucleus obviously 120 minutes after IL-4 stimulation (Figure 3A). Then we tested whether IL-4 and RvD1 affected PPARγ protein levels in nucleus and DNA binding activity. As shown in Figure 3B and C, IL-4 increased PPARγ protein levels in nucleus and DNA binding activity. RvD1 alone failed to activate PPARγ (*P* > 0.05). However, combination of RvD1 and IL-4 further increased PPARγ protein levels in nucleus and DNA binding activity compared to treatment with IL-4 alone, the effects of RvD1 were abolished by pretreatment with Boc-2.

### STAT6 is required for PPARγ activation induced by IL-4 and RvD1

**Figure 4:**
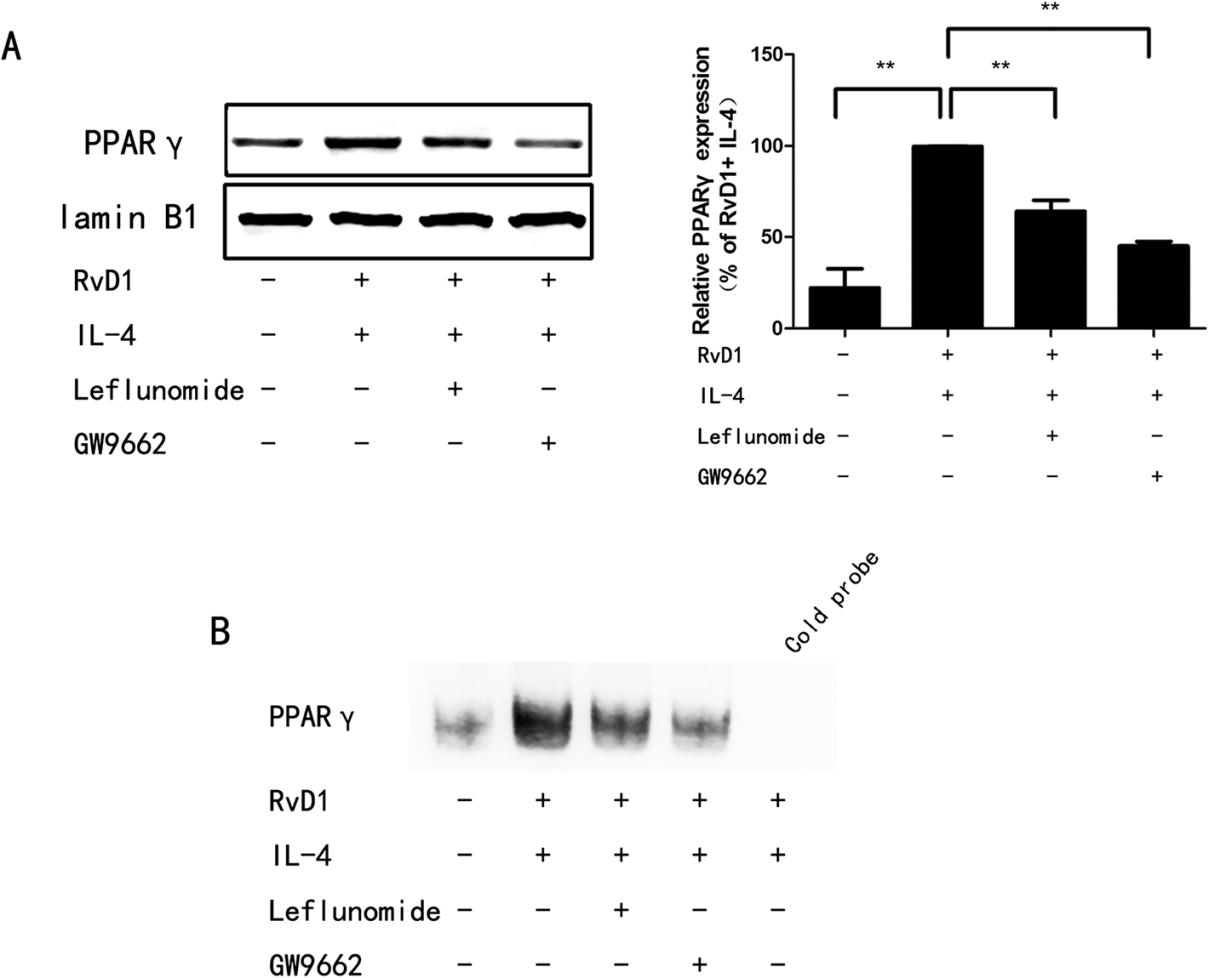
**STAT6 is required for PPARγ activation.** BV-2 cells were treated with leflunomide (10 μM) or GW9662 (1 μM) 30 minutes prior to administration of RvD1 (100 nM), followed by incubation with IL-4 (10 ng/ml) for 120 minutes. PPARγ protein level in nucleus **(A)** and DNA binding activity **(B)** were analyzed using Western blot or electrophoretic mobility shift assay (EMSA). A representative result from three independent experiments is shown. Quantification of PPARγ was normalized by lamin B1. Data are presented as mean ± SEM for three independent experiments. Asterisks indicate statistically significant difference (*P < 0.05, **P < 0.01).

STAT6 is a regulator of PPARγ response; IL-4 induces augmented PPARγ activity through a STAT6-dependent mechanism [11,27]. To identify the role of STAT6 on PPARγ activation induced by IL-4 and RvD1, we used leflunomide (an inhibitor suppressing tyrosine phosphorylation of STAT6 and preventing subsequent DNA binding) and GW9662 (a PPARγ antagonist) [26,28]. One hundred micromoles of leflunomide or 1 μM GW9662 was administrated 30 minutes prior to treatment with RvD1, PPARγ expression and DNA binding activity were analyzed. Consistent with GW9662, leflunomide decreased PPARγ protein levels in nucleus and DNA binding activity in IL-4 and RvD1 treated BV-2 cells (Figure 4).

### STAT6 and PPARγ are essential for induction of Arg1 and Ym1 by IL-4 and RvD1

**Figure 5:**
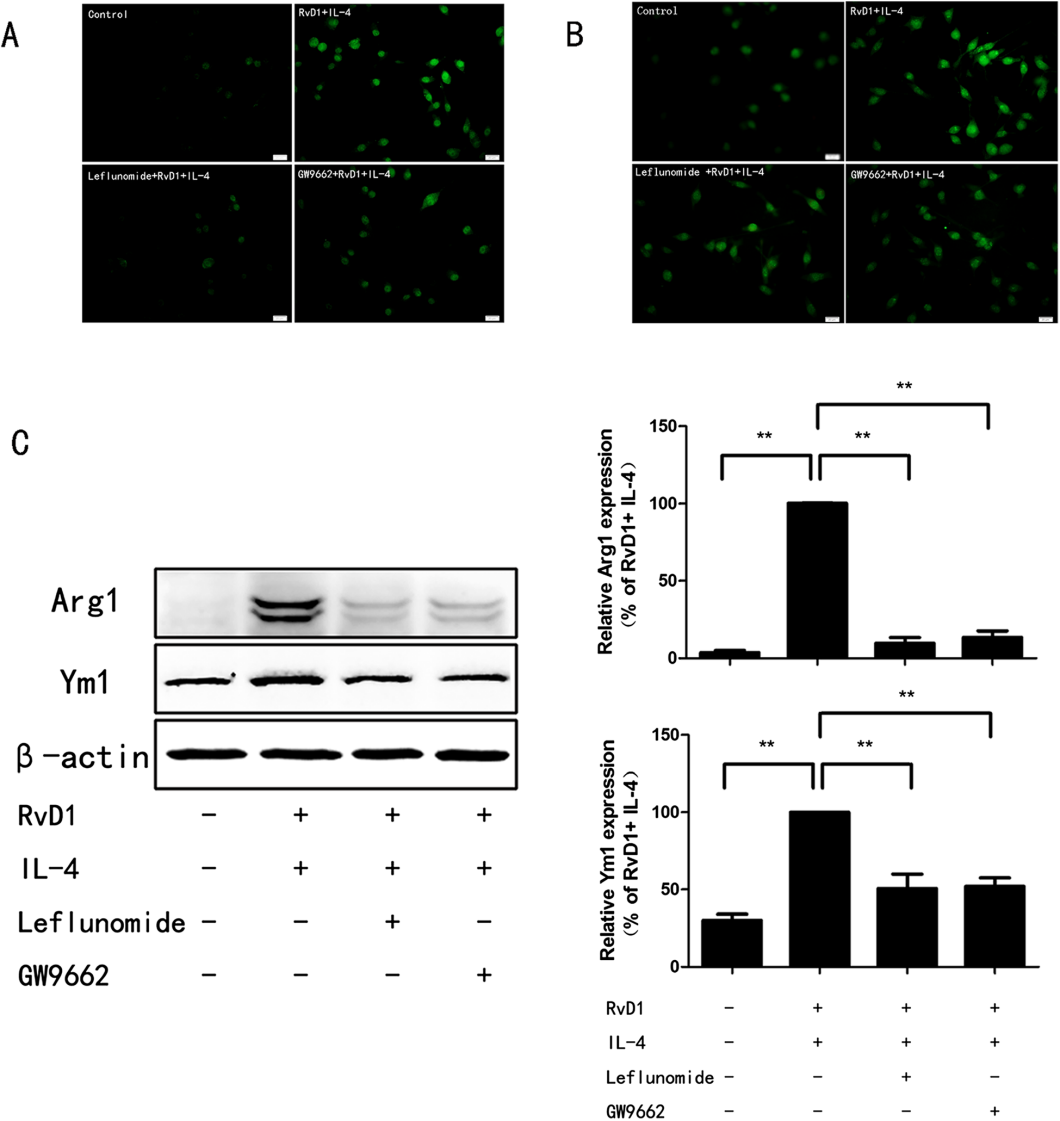
**Inhibition of STAT6 and PPARγ impacts microglia alternative activation induced by IL-4 and RvD1.** BV-2 cells were treated with leflunomide (10 μM) or GW9662 (1 μM) 30 minutes prior to administration of RvD1 (100 nM) and then stimulated with IL-4 (10 ng/ml) for 24 hours. Immunofluorescence assay for Arg1 **(A)** and Ym1 **(B)** indicated that the induction of Arg1 and Ym1 by RvD1 and IL-4 was disappeared when blocking STAT6 or PPARγ signaling pathway. Scale bars indicate 20 μm. **(C)** The protein levels of Arg1 and Ym1 were measured by Western blot. A representative result from three independent experiments is shown. Quantification for Arg1 and Ym1 was normalized by β-actin. Data are presented as mean ± SEM for three independent experiments. Asterisks indicate statistically significant difference (*P < 0.05, **P < 0.01).

RvD1 promotes IL-4-induced STAT6 and PPARγ activation, these facts suggest, but do not prove, that STAT6 and PPARγ are required for the effects of RvD1 and IL-4 on Arg1 and Ym1 induction. To directly test the effects of STAT6 and PPARγ, BV-2 cells were pretreated with 100 μM leflunomide or 1 μM GW9662 for 30 minutes to inhibit STAT6 or PPARγ signaling pathway and stimulated with RvD1 and IL-4 for 24 hours. Immunofluorescence and Western blot were performed to determine Arg1 and Ym1 expression. As expected, Arg1 and Ym1 induction by RvD1 and Ym1 was markedly blunted by inhibition of STAT6 and PPARγ (Figure 5).

## Discussion

In this study, we provide the first evidence that RvD1 enhances the IL-4-induced M2 polarization in BV-2 microglial cells. Pretreatment with RvD1 was able to increase IL-4-induced expression of two major alternative activation markers, Arg1 and Ym1. In addition, RvD1 potentiated the effects of IL-4 on STAT6 and PPARγ signaling pathways, administering leflunomide or GW9662 before RvD1 and IL-4 blunted the induction of Arg1 and Ym1. These results suggest that RvD1 can promote IL-4-induced M2 polarization in microglial cells via STAT6 and PPARγ signaling pathways.

So far, two receptors for RvD1 have been indentified, ALX/FPR2 and an orphan, GPR32. RvD1 activates ALX and GPR32 with high affinity (EC_50_ approximately 1.2 pM for ALX/FPR2 and 8.8 pM for GPR32) [18]. Interestingly, on human polymorphonuclear leukocytes, low concentration of RvD1 (1 nM) appear sensitive to GPR32 blockade, while responses to high concentration (10 nM) are ALX-dependent [29]. Our findings demonstrated that RvD1 promoted IL-4-induced microglia alternative activation with high concentrations (10 nM and 100 nM). Moreover, in murine physiology, we did not identify the murine orthologs of human GPR32, while mouse ALX has been confirmed to express in neuron, astrocyte, microglia, neutrophils, macrophage and monocyte, as well as BV-2 cells [21,30]. Boc-2 is an ALX/FPR2 antagonist, and can block the ability of RvD1 [31]. Thus, we investigated the receptor dependency of RvD1 in microglia M2 polarization by using Boc-2. Previous studies have shown that activation of ALX by lipoxin A4, RvD1, BML-111 or other agonists have potential anti-inflammatory and pro-resolving effects in a rat brain ischemia-reperfusion injury model [32,33] and other inflammatory disease models [34-37]. These findings suggest that RvD1 could modulate the inflammatory reaction in CNS diseases.

Microglia exhibit various functions at different stages in life or in response to different pathological situations [6]. The activation states of microglia are plastic and flexible, both blunting classical activation and promoting alternative activation are involving in anti-inflammatory and pro-resolving process. Here, we used Arg1 and Ym1 as markers for microglia alternative activation. Expression of Arg1 in microglia is induced by Th2 cytokines [38]. Arg1 can reduce NO production, and it is also involved in wound healing, fibrosis and supporting neuron survival [39-41]. Apart from Arg1, Ym1 is another marker for alternative activation [42]. Ym1 is considered to be associated with tissue remodeling and regulating inflammation [43]. First, we examined the effect of IL-4 and RvD1 on Arg1 and Ym1 expression. According with previous studies, our results showed that expression of Arg1 and Ym1 was low in unstimulated BV-2 microglial cells, which was enhanced by stimulation with 10ng/ml IL-4 [7,44]. Titos *et al*. reported that RvD1 and its precursor DHA promoted resolution by eliciting macrophage M2 polarization [20]. In our study, treatment with RvD1 alone had no effect on Arg1 and Ym1 expression in microglia, while pretreatment with RvD1 enhanced the effect of IL-4 on Arg1 and Ym1 expression. This indicated that RvD1 may be possible to promote resolution of neuroinflammation by regulating microglia polarization. The effect of RvD1 was receptor-dependent as it was reversed by the use of ALX antagonist Boc-2.

**Figure 6:**
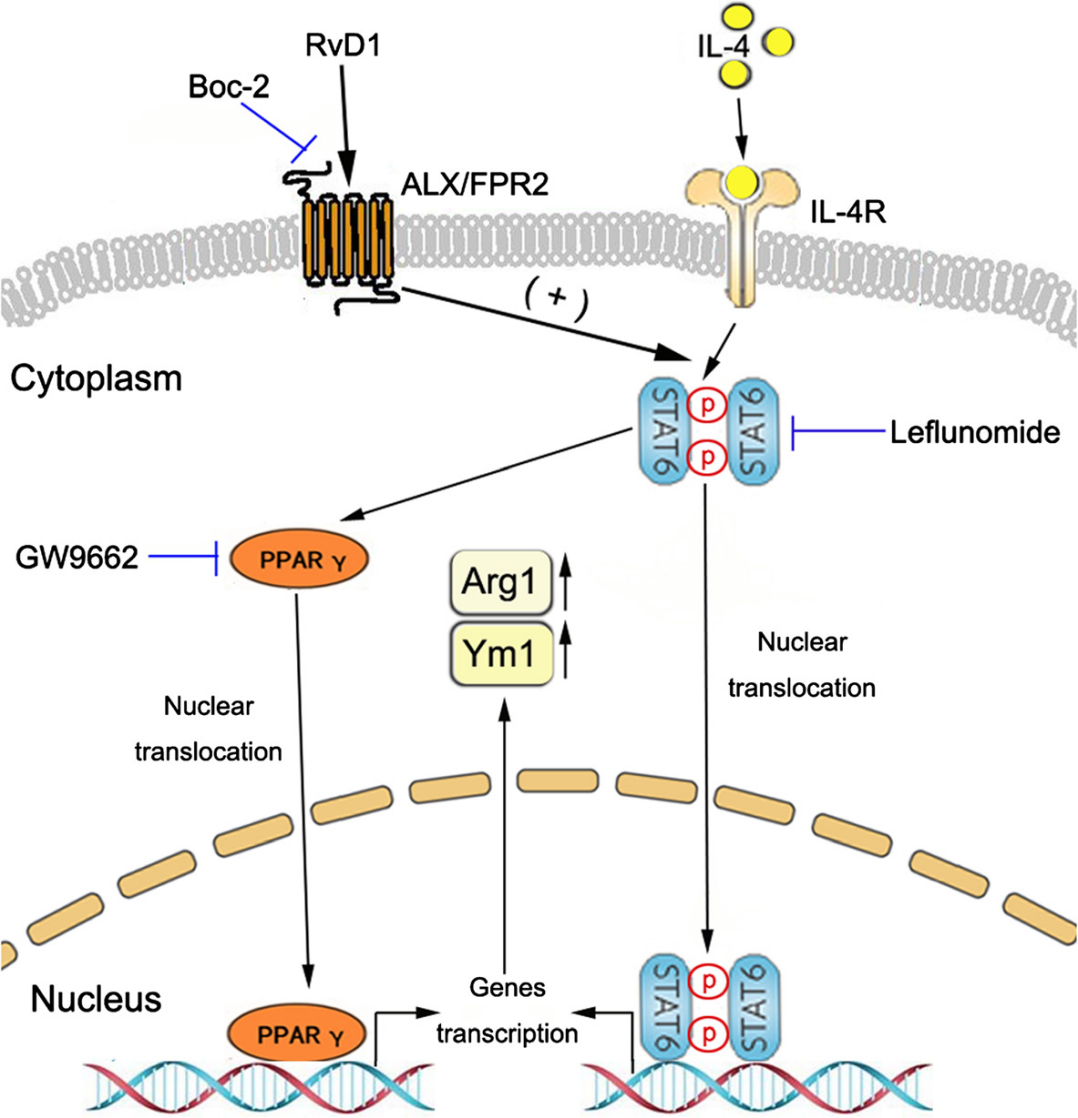
**Schematic representation of the RvD1 contribution to microglia alternative activation.** RvD1 promotes IL-4-induced microglia alternative activation by increasing Arg1 and Ym1 expression via STAT6/PPARγ signaling pathway.

The synergistic effect of RvD1 and IL-4 on Arg1 and Ym1 expression might be explained by RvD1 potentiating IL-4-induced STAT6 and PPARγ activation. IL-4-induced expression of Arg1 and Ym1 depends on activation of STAT6. In response to IL-4, STAT6 is phosphorylated, translates into the nucleus and binds to consensus sites at the Jmjd3 promoter, then regulates transcriptional activation of M2 marker genes [45]. STAT6 can also modulate the activity of nuclear receptor PPARγ [11]. PPARγ plays an important role in macrophage/microglia alternative activation; the addition of a PPARγ agonist directly regulates the Arg1 and Ym1 expression [12,14,46,47]. In this study, RvD1 was able to enhance the phosphorylation and DNA binding activity of STAT6 induced by IL-4. Inhibiting STAT6 by leflunomide abolished the induction of Arg1 and Ym1. Moreover, leflunomide inhibited PPARγ translocating into the nucleus and binding to DNA sites, which suggested that STAT6 was required for PPARγ activation induced by RvD1 and IL-4. Consistent with STAT6, the activity of PPARγ induced by RvD1 and IL-4 was greater than stimulating with IL-4 alone. A previous study reported that activation of PPARγ by pioglitazone promoted microglia M2 polarization by increasing Arg1 and Ym1 expression [14]. In our research, the effects of RvD1 and IL-4 on Arg1 and Ym1 were impaired by using PPARγ antagonist GW9662, which proved PPARγ was essential for RvD1 and IL-4-induced microglia alternative activation. These findings suggested that RvD1 enhanced IL-4-induced microglia alternative activation depending on the STAT6/PPARγ signaling pathway (Figure 6).

## Conclusions

Our results show that RvD1 promotes IL-4-induced microglia alternative activation. A possible mechanism relates to activation of STAT6 and PPARγ signaling pathways. Given the anti-inflammatory and pro-resolving effects of RvD1, it may be a potential treatment for CNS diseases involving neuroinflammation.

## Abbreviations

ALX: lipoxin A4 receptor; Arg1: arginase 1; Boc-2: butyloxycarbonyl-Phe-Leu-Phe-Leu-Phe; BSA: bovine serum albumin; CNS: central nervous system; DAPI: 6-diamidino-2-phenylindole; DHA: docosahexaenoic acid; DMEM: Dulbecco’s modified Eagle’s medium; EMSA: electrophoretic Mobility Shift Assay; EPA: eicosapentaenoic acid; Fizz1: found in inflammatory zone 1; FPR2: formyl peptide receptor 2; GPR 32: G protein coupling receptor 32; IL: interleukin; LPS: lipopolysaccharide; NO: nitric oxide; PBS: phosphate-buffered saline; RvD1: resolvin D1; PPARγ: peroxisome proliferator-activated receptor gamma; RIPA: radioimmune precipitation assay buffer; ROS: reactive oxygen species; STAT6: signal transducer and activators of transcription 6; TBE: Tris-borate-EDTA; TBST: Tris-buffered saline containing 0.05% Tween-20; TNF: tumour necrosis factor; Ym1: chitinase 3-like 3.

## Competing interests

The authors declare that they have no competing interests.

## Authors’ contributions

LYL and YW designed the experiments. LYL, YW, WJX and LMS performed the experiments. LYL and YW analyzed the data. YPW, JW and LP provided useful advice and reviewed the manuscript. SYY and YS supervised the experimental work. YS conceived the study, participated in its design and coordination, and wrote the manuscript. All authors of this paper have read and approved the final version the manuscript.

## References

[B1] Nimmerjahn A Kirchhoff F Helmchen F Resting microglial cells are highly dynamic surveillants of brain parenchyma *in vivo* Science 2005 308 1314 1318 10.1126/science.1110647 15831717

[B2] Nelson PT Soma LA Lavi E Microglia in diseases of the central nervous system Ann Med 2002 34 491 500 10.1080/078538902321117698 12553488

[B3] Mosser DM The many faces of macrophage activation J Leukoc Biol 2003 73 209 212 10.1189/jlb.0602325 12554797

[B4] Hu X Li P Guo Y Wang H Leak RK Chen S Gao Y Chen J Microglia/macrophage polarization dynamics reveal novel mechanism of injury expansion after focal cerebral ischemia Stroke 2012 43 3063 3070 10.1161/STROKEAHA.112.659656 22933588

[B5] Lee DC Ruiz CR Lebson L Selenica ML Rizer J Hunt JB Jr Rojiani R Reid P Kammath S Nash K Dickey CA Gordon M Morgan D Aging enhances classical activation but mitigates alternative activation in the central nervous system Neurobiol Aging 2013 34 1610 1620 10.1016/j.neurobiolaging.2012.12.014 23481567 PMC3652232

[B6] Boche D Perry VH Nicoll JA Review: activation patterns of microglia and their identification in the human brain Neuropathol Appl Neurobiol 2013 39 3 18 10.1111/nan.12011 23252647

[B7] Varnum MM Ikezu T The classification of microglial activation phenotypes on neurodegeneration and regeneration in Alzheimer’s disease brain Arch Immunol Ther Exp (Warsz) 2012 60 251 266 10.1007/s00005-012-0181-2 22710659 PMC4429536

[B8] Gordon S Martinez FO Alternative activation of macrophages: mechanism and functions Immunity 2010 32 593 604 10.1016/j.immuni.2010.05.007 20510870

[B9] Goenka S Kaplan MH Transcriptional regulation by STAT6 Immunol Res 2011 50 87 96 10.1007/s12026-011-8205-2 21442426 PMC3107597

[B10] Mandal P Pratt BT Barnes M McMullen MR Nagy LE Molecular mechanism for adiponectin-dependent M2 macrophage polarization: link between the metabolic and innate immune activity of full-length adiponectin J Biol Chem 2011 286 13460 13469 10.1074/jbc.M110.204644 21357416 PMC3075692

[B11] Szanto A Balint BL Nagy ZS Barta E Dezso B Pap A Szeles L Poliska S Oros M Evans RM Barak Y Schwabe J Nagy L STAT6 transcription factor is a facilitator of the nuclear receptor PPARgamma-regulated gene expression in macrophages and dendritic cells Immunity 2010 33 699 712 10.1016/j.immuni.2010.11.009 21093321 PMC3052437

[B12] Odegaard JI Ricardo-Gonzalez RR Goforth MH Morel CR Subramanian V Mukundan L Red Eagle A Vats D Brombacher F Ferrante AW Chawla A Macrophage-specific PPARgamma controls alternative activation and improves insulin resistance Nature 2007 447 1116 1120 10.1038/nature05894 17515919 PMC2587297

[B13] Jiang Q Heneka M Landreth GE The role of peroxisome proliferator-activated receptor-gamma (PPARgamma) in Alzheimer’s disease: therapeutic implications CNS Drugs 2008 22 1 14 10.2165/00023210-200822010-00001 18072811

[B14] Mandrekar-Colucci S Karlo JC Landreth GE Mechanisms underlying the rapid peroxisome proliferator-activated receptor-gamma-mediated amyloid clearance and reversal of cognitive deficits in a murine model of Alzheimer’s disease J Neurosci 2012 32 10117 10128 10.1523/JNEUROSCI.5268-11.2012 22836247 PMC3433721

[B15] Seki H Sasaki T Ueda T Arita M Resolvins as regulators of the immune system ScientificWorldJournal 2010 10 818 831 20454764 10.1100/tsw.2010.72 PMC5763813

[B16] Hong S Gronert K Devchand PR Moussignac RL Serhan CN Novel docosatrienes and 17S-resolvins generated from docosahexaenoic acid in murine brain, human blood, and glial cells. Autacoids in anti-inflammation J Biol Chem 2003 278 14677 14687 10.1074/jbc.M300218200 12590139

[B17] Rogerio AP Haworth O Croze R Oh SF Uddin M Carlo T Pfeffer MA Priluck R Serhan CN Levy BD Resolvin D1 and aspirin-triggered resolvin D1 promote resolution of allergic airways responses J Immunol 2012 189 1983 1991 10.4049/jimmunol.1101665 22802419 PMC3534750

[B18] Krishnamoorthy S Recchiuti A Chiang N Yacoubian S Lee CH Yang R Petasis NA Serhan CN Resolvin D1 binds human phagocytes with evidence for proresolving receptors Proc Natl Acad Sci U S A 2010 107 1660 1665 10.1073/pnas.0907342107 20080636 PMC2824371

[B19] Krishnamoorthy S Recchiuti A Chiang N Fredman G Serhan CN Resolvin D1 receptor stereoselectivity and regulation of inflammation and proresolving microRNAs Am J Pathol 2018–2027 2012 180 10.1016/j.ajpath.2012.01.028 PMC3349829 22449948

[B20] Titos E Rius B Gonzalez-Periz A Lopez-Vicario C Moran-Salvador E Martinez-Clemente M Arroyo V Claria J Resolvin D1 and its precursor docosahexaenoic acid promote resolution of adipose tissue inflammation by eliciting macrophage polarization toward an M2-like phenotype J Immunol 2011 187 5408 5418 10.4049/jimmunol.1100225 22013115

[B21] Wang YP Wu Y Li LY Zheng J Liu RG Zhou JP Yuan SY Shang Y Yao SL Aspirin-triggered lipoxin A4 attenuates LPS-induced pro-inflammatory responses by inhibiting activation of NF-kappaB and MAPKs in BV-2 microglial cells J Neuroinflammation 2011 8 95 10.1186/1742-2094-8-95 21831303 PMC3162900

[B22] Wu Y Zhai H Wang Y Li L Wu J Wang F Sun S Yao S Shang Y Aspirin-triggered lipoxin A(4) attenuates lipopolysaccharide-induced intracellular ROS in BV2 microglia cells by inhibiting the function of NADPH oxidase Neurochem Res 2012 37 1690 1696 10.1007/s11064-012-0776-3 22552474

[B23] Ye XH Wu Y Guo PP Wang J Yuan SY Shang Y Yao SL Lipoxin A4 analogue protects brain and reduces inflammation in a rat model of focal cerebral ischemia reperfusion Brain Res 2010 1323 174 183 20138164 10.1016/j.brainres.2010.01.079

[B24] Pourcet B Pineda-Torra I Transcriptional regulation of macrophage arginase 1 expression and its role in atherosclerosis Trends Cardiovasc Med 2013 23 143 152 10.1016/j.tcm.2012.10.003 23375628

[B25] Welch JS Escoubet-Lozach L Sykes DB Liddiard K Greaves DR Glass CK TH2 cytokines and allergic challenge induce Ym1 expression in macrophages by a STAT6-dependent mechanism J Biol Chem 2002 277 42821 42829 10.1074/jbc.M205873200 12215441

[B26] Gallardo-Soler A Gomez-Nieto C Campo ML Marathe C Tontonoz P Castrillo A Corraliza I Arginase I induction by modified lipoproteins in macrophages: a peroxisome proliferator-activated receptor-gamma/delta-mediated effect that links lipid metabolism and immunity Mol Endocrinol 2008 22 1394 1402 10.1210/me.2007-0525 18323470 PMC5419540

[B27] Vats D Mukundan L Odegaard JI Zhang L Smith KL Morel CR Wagner RA Greaves DR Murray PJ Chawla A Oxidative metabolism and PGC-1beta attenuate macrophage-mediated inflammation Cell Metab 2006 4 13 24 10.1016/j.cmet.2006.05.011 16814729 PMC1904486

[B28] Siemasko K Chong AS Jack HM Gong H Williams JW Finnegan A Inhibition of JAK3 and STAT6 tyrosine phosphorylation by the immunosuppressive drug leflunomide leads to a block in IgG1 production J Immunol 1998 160 1581 1588 9469413

[B29] Norling LV Dalli J Flower RJ Serhan CN Perretti M Resolvin D1 limits polymorphonuclear leukocyte recruitment to inflammatory loci: receptor-dependent actions Arterioscler Thromb Vasc Biol 1970–1978 2012 32 10.1161/ATVBAHA.112.249508 PMC3401489 22499990

[B30] Ye RD Boulay F Wang JM Dahlgren C Gerard C Parmentier M Serhan CN Murphy PM International Union of Basic and Clinical Pharmacology. LXXIII. Nomenclature for the formyl peptide receptor (FPR) family Pharmacol Rev 2009 61 119 161 10.1124/pr.109.001578 19498085 PMC2745437

[B31] Odusanwo O Chinthamani S McCall A Duffey ME Baker OJ Resolvin D1 prevents TNF-alpha-mediated disruption of salivary epithelial formation Am J Physiol Cell Physiol 2012 302 C1331 C1345 10.1152/ajpcell.00207.2011 22237406 PMC3361948

[B32] Wu Y Wang YP Guo P Ye XH Wang J Yuan SY Yao SL Shang Y A lipoxin A4 analog ameliorates blood–brain barrier dysfunction and reduces MMP-9 expression in a rat model of focal cerebral ischemia-reperfusion injury J Mol Neurosci 2012 46 483 491 10.1007/s12031-011-9620-5 21845429

[B33] Wu Y Ye XH Guo PP Xu SP Wang J Yuan SY Yao SL Shang Y Neuroprotective effect of lipoxin A4 methyl ester in a rat model of permanent focal cerebral ischemia J Mol Neurosci 2010 42 226 234 10.1007/s12031-010-9355-8 20401639

[B34] Cattaneo F Parisi M Ammendola R Distinct signaling cascades elicited by different formyl Peptide receptor 2 (FPR2) agonists Int J Mol Sci 2013 14 7193 7230 10.3390/ijms14047193 23549262 PMC3645683

[B35] Gong J Guo S Li HB Yuan SY Shang Y Yao SL BML-111, a lipoxin receptor agonist, protects haemorrhagic shock-induced acute lung injury in rats Resuscitation 2012 83 907 912 10.1016/j.resuscitation.2011.12.035 22245750

[B36] Li HB Wang GZ Gong J Wu ZY Guo S Li B Liu M Ji YD Tang M Yuan SY Yao SL BML-111 attenuates hemorrhagic shock-induced acute lung injury through inhibiting activation of mitogen-activated protein kinase pathway in rats J Surg Res 2013 183 710 719 10.1016/j.jss.2013.03.007 23558258

[B37] Shang Y Jiang YX Ding ZJ Shen AL Xu SP Yuan SY Yao SL Valproic acid attenuates the multiple-organ dysfunction in a rat model of septic shock Chin Med J (Engl) 2010 123 2682 2687 21034653

[B38] Pauleau AL Rutschman R Lang R Pernis A Watowich SS Murray PJ Enhancer-mediated control of macrophage-specific arginase I expression J Immunol 2004 172 7565 7573 15187136 10.4049/jimmunol.172.12.7565

[B39] Morris SM Jr Enzymes of arginine metabolism J Nutr 2004 134 2743S 2747S discussion 2765S-2767S 15465778 10.1093/jn/134.10.2743S

[B40] Iniesta V Gomez-Nieto LC Molano I Mohedano A Carcelen J Miron C Alonso C Corraliza I Arginase I induction in macrophages, triggered by Th2-type cytokines, supports the growth of intracellular Leishmania parasites Parasite Immunol 2002 24 113 118 10.1046/j.1365-3024.2002.00444.x 11982856

[B41] Ma TC Campana A Lange PS Lee HH Banerjee K Bryson JB Mahishi L Alam S Giger RJ Barnes S Morris SM Jr Willis DE Twiss JL Filbin MT Ratan RR A large-scale chemical screen for regulators of the arginase 1 promoter identifies the soy isoflavone daidzeinas a clinically approved small molecule that can promote neuronal protection or regeneration via a cAMP-independent pathway J Neurosci 2010 30 739 748 10.1523/JNEUROSCI.5266-09.2010 20071539 PMC3554247

[B42] Colton CA Mott RT Sharpe H Xu Q Van Nostrand WE Vitek MP Expression profiles for macrophage alternative activation genes in AD and in mouse models of AD J Neuroinflammation 2006 3 27 10.1186/1742-2094-3-27 17005052 PMC1609108

[B43] Giannetti N Moyse E Ducray A Bondier JR Jourdan F Propper A Kastner A Accumulation of Ym1/2 protein in the mouse olfactory epithelium during regeneration and aging Neuroscience 2004 123 907 917 10.1016/j.neuroscience.2003.09.024 14751284

[B44] Zhou X Spittau B Krieglstein K TGFbeta signalling plays an important role in IL4-induced alternative activation of microglia J Neuroinflammation 2012 9 210 10.1186/1742-2094-9-210 22947253 PMC3488564

[B45] Ishii M Wen H Corsa CA Liu T Coelho AL Allen RM Carson WF Cavassani KA Li X Lukacs NW Hogaboam CM Dou Y Kunkel SL Epigenetic regulation of the alternatively activated macrophage phenotype Blood 2009 114 3244 3254 10.1182/blood-2009-04-217620 19567879 PMC2759649

[B46] Cuartero MI Ballesteros I Moraga A Nombela F Vivancos J Hamilton JA Corbi AL Lizasoain I Moro MA N2 neutrophils, novel players in brain inflammation after stroke: modulation by the PPARgamma agonist rosiglitazone Stroke 2013 44 3498 3508 10.1161/STROKEAHA.113.002470 24135932

[B47] Bouhlel MA Derudas B Rigamonti E Dievart R Brozek J Haulon S Zawadzki C Jude B Torpier G Marx N Staels B Chinetti-Gbaguidi G PPARgamma activation primes human monocytes into alternative M2 macrophages with anti-inflammatory properties Cell Metab 2007 6 137 143 10.1016/j.cmet.2007.06.010 17681149

